# Evaluating growth response of broiler chickens fed diets supplemented with synthetic DL-methionine or DL-hydroxy methionine: a meta-analysis

**DOI:** 10.1016/j.psj.2022.101762

**Published:** 2022-02-02

**Authors:** M.E. Uddin, Henk J. van Lingen, Paula G. da Silva-Pires, Dolores I. Batonon-Alavo, Friedrich Rouffineau, Ermias Kebreab

**Affiliations:** ⁎ Department of Animal Science, University of California, Davis, CA 95616, USA; † Dairy and Food Science Department, South Dakota State University, Brookings, SD 57007, USA; ‡ Laboratory of Systems and Synthetic Biology, Wageningen University & Research, P.O. Box 8033, 6700 EJ Wageningen, the Netherlands; § Department of Animal Science, Universidade Federal do Rio Grande do Sul, Brazil; # Adisseo France SAS, F-03630 Malicorne, France

**Keywords:** average daily gain, methionine analogue, sulfur amino acid, Bayesian analysis

## Abstract

Methionine (**Met**) is the first limiting amino acid in corn and soybean meal-based diets (containing L-Met) in broiler chickens, which are often supplemented with synthetic DL-Met or DL-Hydroxy Met (**OH-Met).** Our objective was to quantitatively assess the efficacy of synthetic Met sources and determine differences in growth rate of broilers fed at or below requirements in response to Met intake. A systematic literature search resulted in building a database containing 480 treatment means from 39 articles published between 1985 and 2019 globally. The database was divided into starter, grower, and finisher subsets based on the age of the broilers. For each subset, linear-plateau and quadratic-plateau models were fitted to determine Met or sulfur amino acid (**SAA**; Met + Cysteine) requirements using average daily gain as a response variable. For each phase, 4 new subsets were obtained by only retaining records with digestible Met or SAA intake at or below requirement by linear-plateau or quadratic-plateau models. Then, a linear model (without plateau) was fitted for all new subsets for each rearing phase using supplemental digestible synthetic Met or SAA intake (basal Met intake was subtracted from total Met intake) as independent variables. The basal diet was made of only raw materials without supplementation of any synthetic Met source. Finally, the models were extended to evaluate source of synthetic Met effects on the slope parameter. At all stages of model fitting, the inclusion of a random study effect was evaluated for each parameter. All models were fitted within a Bayesian framework, for which minimally informative priors were used. The best models, that is, the most accurate inclusion of random effects, were selected based on at least 10-point difference in leave-one-out cross-validation information criterion. Model selection criteria did not consistently favor either of the linear- and quadratic-plateau models to determine Met or SAA requirements across broiler growth phases. Extending models with covariates (e.g., dietary energy and amino acids) did not improve any model fit. Body weight gain response of broiler chickens to the 2 sources was not different when fed at or below Met requirements for any of the growth phases.

## INTRODUCTION

Methionine (**Met**) is the first limiting amino acid in corn and soybean meal-based broiler diets for broiler chickens. Methionine plays a key role in broilers, primarily feather growth and protein synthesis (Bunchasak, 2009). Diets of non-ruminant animals are often supplemented with Met in multiple forms such as dry DL-Met or liquid DL-hydroxy-Met (**OH-Met**) also known as 2-hydroxy-4-(methylthio)butanoate (**HMTBA**) or methionine hydroxy analogue – free acid (**MHA-FA**). However, animals can only utilize L-Met for protein synthesis. The other forms of Met act as precursors of L-Met which must be converted by the animals to L-Met to be utilized (Dibner and Ivey, 1992; Martín-Venegas et al., 2006). Thus, relative biological efficacy of DL-Met in comparison with OH-Met is a relevant characteristic for feed formulation and cost-effective purchase (Sauer et al., 2008).

For broilers, few Met requirements have been proposed (NRC, 1994; Rostagno et al., 2011, 2017). The Met requirement may vary depending on various characteristics such as age (Oliveira-Neto, 2014). Differences may also arise due to the choice of the models used to determine the requirements (Pomar et al., 2003), or birds’ response variable (weight gain, feed conversion, or breast meat yield) used to estimate the requirement (Oliveira-Neto, 2014). Although multiple investigations reported on Met requirement, there is no consensus on which value to use.

Several models are available in the literature for predicting the growth response of broilers fed different Met precursors (Fattori et al., 1991; Cooper and Washburn, 1998; Aerts et al., 2003) but those that compare the relative biological efficacy of DL-Met with OH-Met are scarce (Kratzer and Littell, 2006; Vazquez-Anon et al., 2006; Sauer et al., 2008). A meta-analysis that compared Met sources (DL-Met vs. Met-hydroxy-analogue-free-acid) was conducted by Sauer et al. (2008) but only included studies published until 2006. A new meta-analysis based on recent empirical studies in the literature comparing DL-Met and OH-Met is lacking. Furthermore, development in statistical methods and software (packages such as WinBUGS and Stan) has enabled fitting of complex models using Bayesian approach, which can take into account the multiple variances in a single model simultaneously (Sorensen et al., 2016). Therefore, fitting model using Bayesian approach accounting for multiple variances instead of using frequentist approach such as nonlinear regression (Sauer et al., 2008) or multi-exponential regression (Hoehler et al., 2005) will strengthen the comparison between DL-Met and OH-Met. Thus, the objective of this study was to provide a summary of the current literature available on Met sources for broiler chickens and to predict the weight gain response to Met intake using different mathematical growth functions.

## MATERIALS AND METHODS

### Search Strategy and Inclusion Criteria

**Figure 1:**
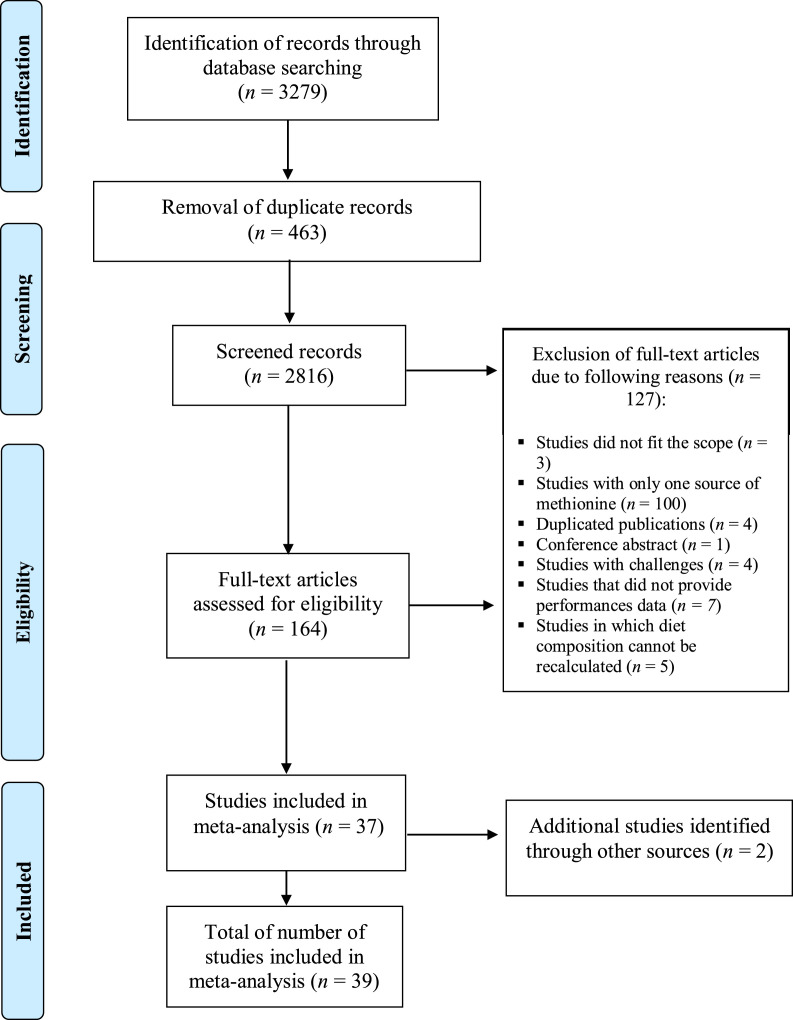
Literature search and selection process following the PRISMA procedure.

A literature search was performed on pre-defined scientific databases including Web of Knowledge, Scopus, and PubMed. The search was performed in each database, using the keywords “methionine OR hydroxy-methionine AND broiler”. To maximize the search completeness, a new search was performed with a larger number of keywords. The new search was performed in each base plus SciELO, using the keywords: “hydroxy analog OR OH-Met OR methionine hydroxy analog OR 2-hydroxy-4-methylthio-butanoic acid AND broiler OR chickens* OR poultry”. A total of 3,279 publications were identified through the literature search. In the next step, after removing duplicate papers, the results found in each database were exported to an excel file and the title and abstract of the studies were examined to exclude irrelevant articles (Figure 1). The full texts of the remaining publications were read, and the growth response, feed and nutrient intake and dietary nutrient composition of the manuscripts were organized for possible classification of eligibility.

The papers were selected using the following inclusion criteria: a) articles that compared DL-Met and OH-Met where studies indicated the source and levels of the Met, b) articles with detailed description of diet composition, c) articles that reported at least 2 of these variables: feed intake, weight gain, or feed conversion ratio (**FCR**), and d) articles that were published in English, Portuguese or Spanish. Additionally, studies where interactions other than the Met effects such as studies with animals under thermo-stressed conditions were excluded. Studies must have been published in peer-reviewed journals. Finally, a total of 39 studies were considered eligible for this meta-analysis. The selection process is shown as PRISMA flowchart according to Moher et al. (2009) in Figure 1.

### Database

The database included 480 records of treatments means from 39 studies conducted from 1985 to 2019 by research entities from Asia: China (*n* = 132 from 6 studies), South Korea (*n* = 14 from 1 study), Thailand (*n* = 15 from 2 studies), Iran (*n* = 8 from 1 study), India (*n* = 6 from 1 study) and Turkey (*n* = 5 from 1 study); Australia (*n* = 5 from 1 study); Europe: Germany (*n* = 58 from 5 studies) the Netherlands (*n* = 45 from 2 studies), Poland (*n* = 9 from 1 study), Spain (*n* = 9 from 1 study), France (*n* = 4 from 1 study); North America: United States (*n* = 88 from 6 studies), Canada (*n* = 7 from 1 study), and Mexico (*n* = 8 from 1 study); and South America: Brazil (*n* = 55 from 8 studies). The list of articles and reports included in the database are given in the supplementary material.

Studies included in this database used broilers from different strains such as Cobb, Ross, Arbor Acres, Indian River, and Shaver Starbro. The database also contained information about the trial (authors, journal, year), experimental design (number of repetitions per treatment, number of animals per repetition, weight range of the broilers), ingredient composition, and nutritional values of the experimental diets and performance information such as ADG, ADFI, and FCR.

Some studies did not report dietary nutrient composition. Thus, the nutrient composition of all diets was recalculated using the Practical Program for Formulation of Rations (**PPFR**, Garcia-Neto, 2008) to get a complete and consistent (i.e., calculated with similar method) dietary nutrient composition. All energy and nutrients available in the software were considered including AME, CP, amino acids, and some minerals. Additionally, the daily nutrient intake was estimated by multiplying the daily feed intake with respective dietary nutrient concentration.

Based on the age of the birds, the broiler database was subsetted into starter (≤21 d of age at the end of the experiment and ≤16 d as average experimental age), grower (<21 and >21 days of age at the start and end of the experiment respectively, and >16 d of average age) and finisher (≥ 21 d of age at the start of the experiment) phases. The relationships between predetermined variables (e.g., ADG vs. animal age, ADG vs. feed intake, ADG vs. Met dose, ADG vs. Met intake) were visually assessed using scatter plots for each growth phase. This visual exploration resulted in the identification of outliers and next in the removal of the records that belonged to 4 different studies.

### Model Development

#### Estimating Requirement for Digestible Methionine and Sulfur Amino Acid Intake

**Figure 2:**
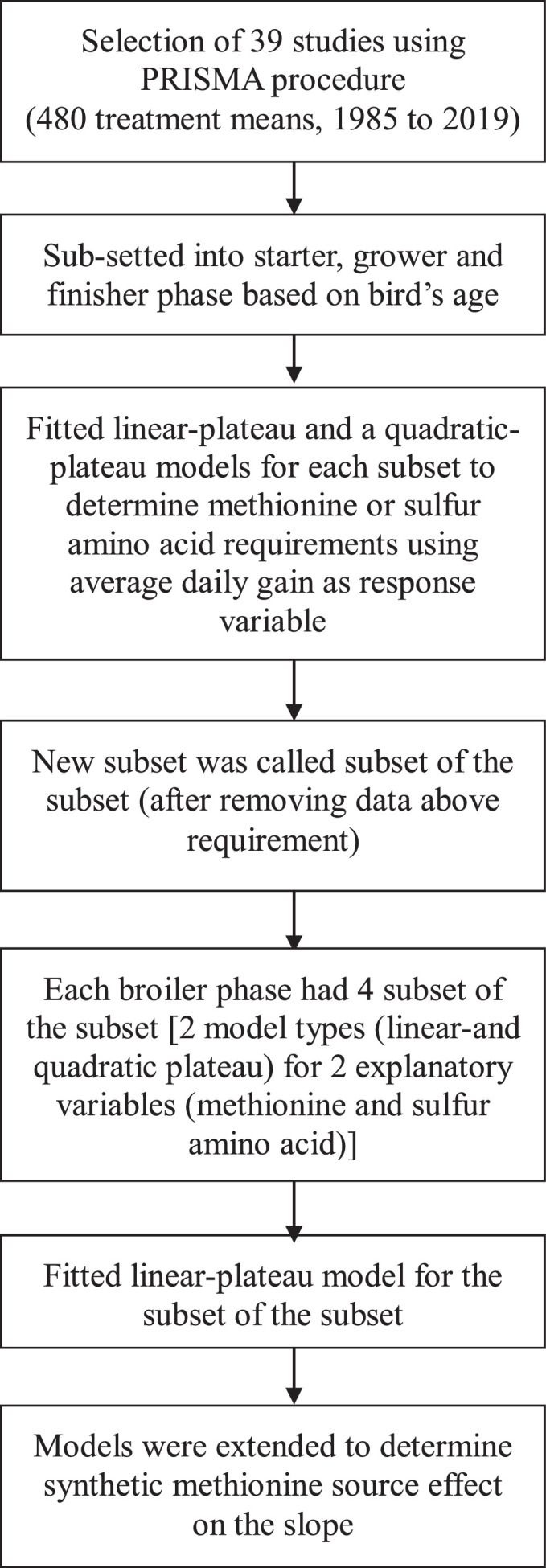
Flow chart showing the Bayesian model fitting steps.

The ADG of the 3 broiler subsets was predicted from digestible Met or digestible sulfur amino acid (**SAA**; i.e., Met+Cysteine) intake as independent variable using a 3-stage hierarchical model (Figure 2). The first stage is the model for the data given the model parameters and variance (Equation 1):

(1) $$y_{ij}=f\left(\theta_{i},x_{ij}\right)+e_{ij}$$

where $y_{ij}$ is the ADG of broiler *j* (*j* = 1, …, *n*) in study *i* (*i* = 1, …, m), $f(\theta_{i},x_{ij})$ is the growth function, $x_{ij}$ is the corresponding digestible Met or SAA intake, $\theta_{i}={\left[\alpha_{i},\beta_{i},\kappa_{i}\right]}^{T}$ is the parameter vector for study *i*, and $e_{ij}$ is the residual error with distribution *N*(0, $\sigma_{e}^{2}$). The growth function per study *i* was represented by linear-plateau function (Equation 2):

(2) $$f\left(\theta_{i},x_{ij}\right)=\alpha_{i}+\beta_{i}\cdot min\left(x_{ij},\;\kappa_{i}\right)$$

a quadratic-plateau function (Equation 3):

(3) $$f\left(\theta_{i},x_{ij}\right)=\alpha_{i}+\beta_{i}\cdot min\left(x_{ij},\;\kappa_{i}\right)+\left(\frac{-\beta_{i}}{2\kappa_{i}}\right)\cdot min\left(x_{ij}^{2},{\kappa_{i}}^{2}\right)$$

and a piecewise linear model function (Equation 4):

(4) $$f\left(\theta_{i},x_{ij}\right)=\alpha_{i}+\beta_{1}x_{ij}+\beta_{2}\cdot max\left(0,x_{ij}-\kappa_{i}\right)$$

$\alpha_{i}$ denote the intercept, $\beta_{i}$ is the slope before breakpoint $\kappa_{i}$. The second stage of the hierarchical model represents the between-study variability (Equation 5):

(5) $$\theta_{i}=\theta +s_{i},$$

with the population parameter vector $\theta ={\left[\alpha ,\beta ,\kappa \right]}^{T}$ and a study-effects vector that contained no more than one study effect for each of the 3 parameters of the linear-plateau and quadratic-plateau models, $s_{i}={\left[a_{i},b_{i},k_{i}\right]}^{T}$. The study effects were distributed according to $s_{i}∼N\left(0,\Psi \right)$, where Ψ is a covariance matrix with maximum dimensions of 3 × 3 and the corresponding diagonals $\sigma_{a}^{2}$, $\sigma_{b}^{2}$ and $\sigma_{k}^{2}$. All off-diagonal elements of Ψ were set at 0, which indicates independent study effects. The third stage of the hierarchical model describes the priors, which were specified as:

(6) $$\left\{ \begin{array}{c} \alpha ,\beta \;∼\;N\left(\mu =0,\;\sigma =100\right)\\ \kappa \;∼\;\text{Unif}\left(\text{min}\left(x_{ij}\right),\;\text{max}\left(x_{ij}\right)\right)\\ \sigma_{a},\sigma_{b},\sigma_{k},\sigma_{e}\;∼\;\text{half}-\text{Cauchy}\left(0,\;5\right) \end{array}\right.$$

**Figure 3:**
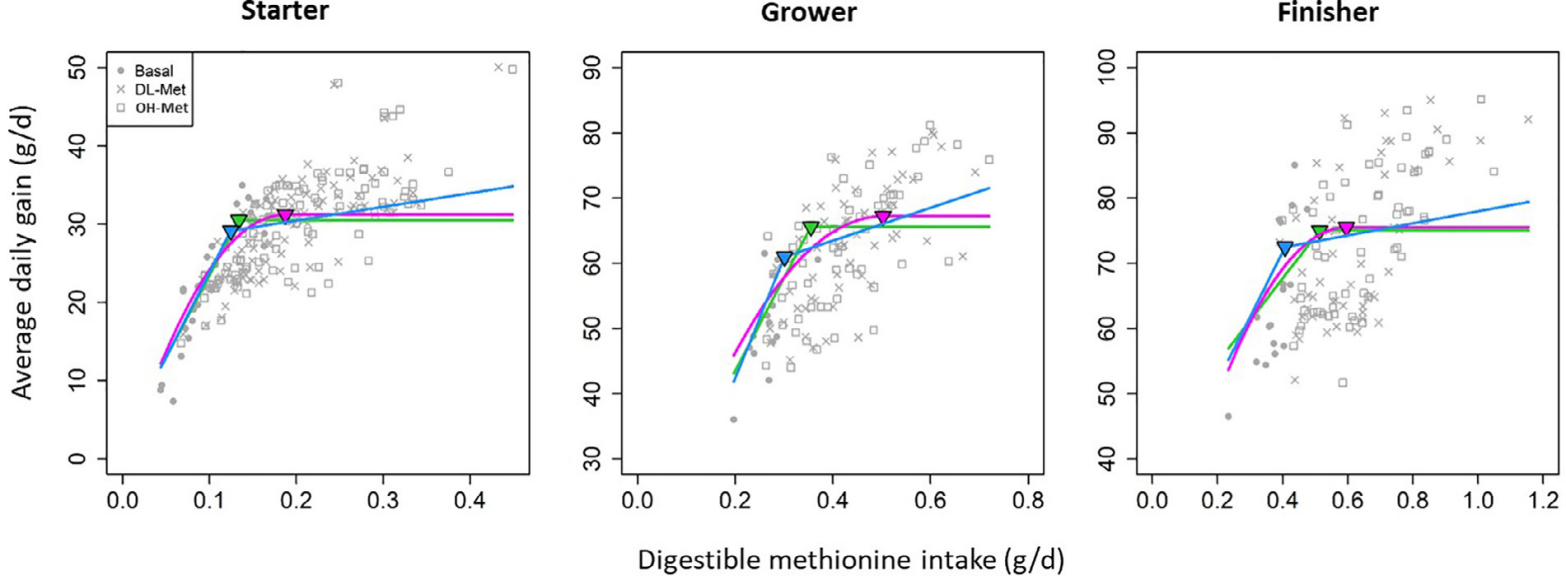
Linear-plateau, quadratic-plateau and piecewise-linear model fits of average daily gain against digestible methionine intake for the starter, grower, and finisher subsets of data. Model parameters are reported in Table 2. These models shown in this figure did not account for the type of Met. Green, pink and blue lines indicate the linear-plateau, quadratic-plateau and piecewise-linear model, respectively.

**Figure 4:**
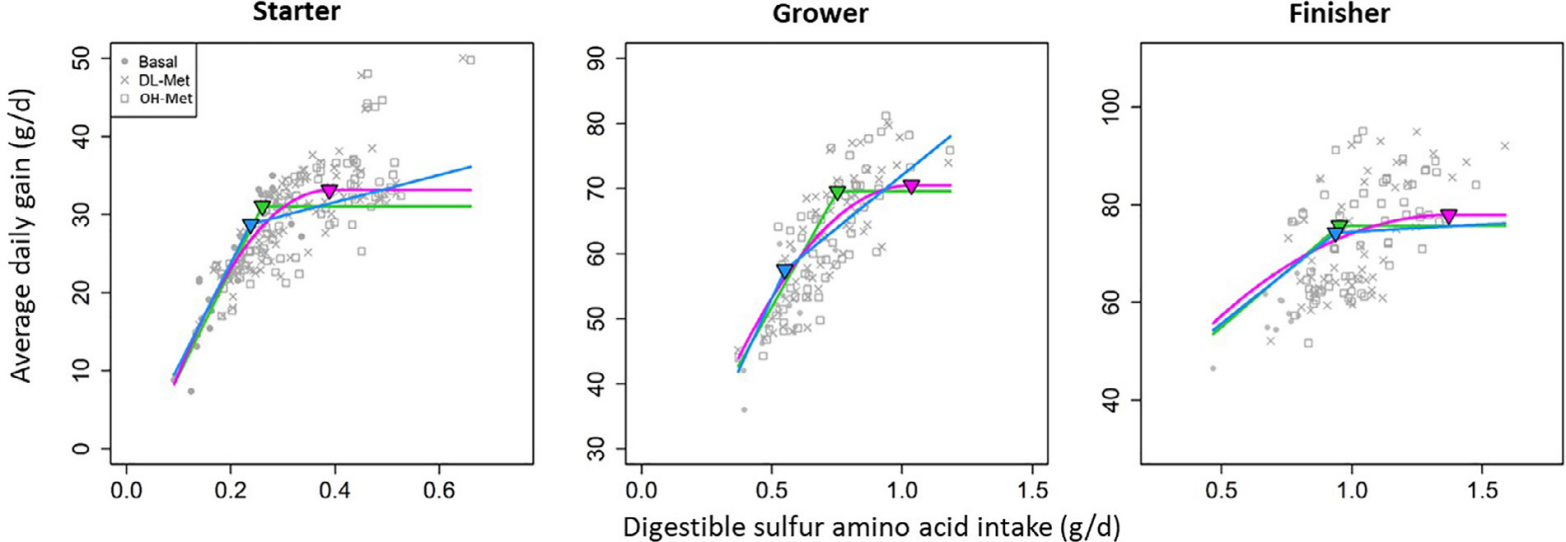
Linear-plateau, quadratic-plateau and piecewise-linear model fits of average daily gain against digestible sulfur amino acid intake using the starter, grower and finisher subsets of data. Model parameters are reported in Table 3. Note that the models shown in this figure did not account for the type of Met. Green, pink and blue lines indicate the linear-plateau, quadratic-plateau, and piecewise-linear model, respectively.

The choice of prior distributions for $\alpha ,\;\beta ,\;\sigma_{a},\;\sigma_{b},\;\sigma_{k}$ and $\sigma_{e}$ was based on the construction of minimally informative priors, whereas the prior distribution for the digestible Met requirement parameter κ constrained to the range of actual digestible Met or SAA intake of birds in each study. Additionally, we also fitted piecewise-linear model which showed poor fits compared to linear-and quadratic plateau models for all phases (Figures 3 and 4). Thus, details of piecewise-linear model were not reported in this study.

The actual inclusion of a random study effect per parameter, that is, the specification of the second stage, was evaluated by fitting the hierarchical model structure to the data with a forward selection procedure. At the start of this procedure, a more parsimonious model was considered that had only one random effect in total, which could apply to any of the three parameters α,β and κ. The best of these three models was selected based on the leave-one-out cross-validation information criterion (**LOOIC**; Vehtari et al., 2017). Subsequently, the selected model was extended with a second random effect on either of the 2 parameters for which no random effect was selected yet. The model with the lowest LOOIC was selected if a 10-point LOOIC decrease was obtained relative to the selected model that had only one random effect. If this criterion was met, the most complex model with a random effect for each of the 3 parameters α,β and κ was evaluated and selected if the LOOIC decreased by 10-points compared to the best model that included 2 of the 3 possible random effects. For every model that was evaluated, 2 chains were run for 30 × 10^3^ iterations with the first 10 × 10^3^ taken as burn-in period. Chains were thinned by a factor of 25, after which convergence was assessed by visually inspecting the chain traces. Upon selecting the random-effects structure per model, this selected model was refitted by running 2 chains for 50 × 10^3^ iterations with the first 20 × 10^3^ taken as burn-in period.

#### Assessing Synthetic Methionine Effect

After fitting the hierarchical model structure using digestible Met or digestible SAA intake as independent variables, the starter, grower, and finisher subsets were further subsetted based on the estimated value of the requirement parameter κ. Only observations with a digestible Met or SAA intake less than or equal to κ that was obtained from the linear-plateau or quadratic-plateau model fitted were retained. This resulted in a maximum of four subsets from the *k* estimated using digestible Met or digestible SAA intake by linear-plateau or quadratic-plateau model for each of starter, grower and finisher subset. Using subsets of a starter, grower or finisher subset, a similar hierarchical model was then used for fitting, but at the first stage the independent variable $x_{ij}$ represented digestible synthetic Met intake instead of total digestible Met intake. In other words, basal digestible Met intake was subtracted from treatment digestible Met intake within study, which then resulted in zero synthetic Met intake for all basal treatments in the database. Furthermore, the growth functions had no plateaus and represented a linear model (Equation 6.1):

(6.1) $$f\left(\theta_{i},x_{ij}\right)=\alpha_{i}+\beta_{i}\cdot x_{ij},$$

where, the intercept parameter $\alpha_{i}$ represents the average daily gain at zero supplementation of synthetic Met source, and $\beta_{i}$ is the slope of the function. The κ parameter of the quadratic function was assigned the maximum values of $x_{ij}$ of the specific subset of a subset used for model fitting, which resulted in the top of the parabolic function at the maximum value of $x_{ij}$. Priors used for the linear model was as described for the linear-plateau model (Equation 5). All other aspects of fitting including the selection of random effects was similar as described for the linear-plateau model. Before fitting these linear models to the subsets of the 3 subsets for starter, grower and finisher broilers, all records associated with a zero dose of synthetic Met were taken twice so that per observation, a zero dose could be assigned to DL-Met and OH-Met. These updated subsets were used for fitting the linear hierarchical models, after which the model was extended for evaluating any synthetic Met effect on the β parameter according to (Equation 7):

(7) $$\beta_{n}=\delta_{n1}z_{n1}+\delta_{n2}z_{n2}$$

with $\left[\begin{array}{c} z_{n1}\\ z_{n2} \end{array}\right]=\left[\begin{array}{c} 1\\ 0 \end{array}\right]$ for DL-Met and $\left[\begin{array}{c} z_{n1}\\ z_{n2} \end{array}\right]=\left[\begin{array}{c} 0\\ 1 \end{array}\right]$ for OH-Met, and $\delta_{n1}$ and $\delta_{n2}$ are the DL-Met and OH-Met main effects, respectively. No synthetic Met effect on α was considered because the zero dose does not contain any synthetic DL-Met or OH-Met. To select random-effects parameters, a 10-points decrease in LOOIC was required for selecting a model with a Met effect on β.

All model simulations were run through the *rstan* (Stan Development Team, 2016) and *loo* packages (Vehtari and Gelman, 2016) in R (version 3.6.3 R Foundation for Statistical Computing, Vienna, Austria).

## RESULTS

### Dietary Information and Performance Data of the Broiler Database

Growth performance and nutrient composition of broiler diets grouped into starter, grower, and finisher are shown in Table 1. As birds grew older, ADG, ADFI and FCR of birds also increased. With increasing age of the birds, dietary energy concentrations increased as typically observed for broiler diets. As expected, the starter phase had greater CP concentrations compared to grower and finisher phases. Similarly, total and digestible Lys and concentrations of other amino acids in the diets decreased with increasing age of the birds. Digestible Met or digestible SAA intake was approximately 3 times the starting value for grower compared to starter and 4 times the starting value for finisher compared to starter phase. Average synthetic Met dose in the diet was similar between grower and finisher phases but slightly greater for starter phase compared to the other two phases.

**Table 1:** Summary of dietary nutrient composition and performance of broilers extracted from the literature.

	Starter (*n* = 221)				Grower (*n* = 126)				Finisher (*n* =133)			
Variables	Mean	SD	Min	Max	Mean	SD	Min	Max	Mean	SD	Min	Max
CP, % of DM	21.6	1.61	18.0	25.7	20.7	2.21	17.5	24.5	19.4	1.33	17.0	22.7
ME, Mcal/kg	2.98	0.116	2.67	3.21	3.01	0.200	2.47	3.46	3.14	0.086	2.94	3.24
Total Lys, %	1.27	0.112	1.10	1.65	1.20	0.113	1.02	1.59	1.13	0.122	1.00	1.49
Digestible Lys, %	1.15	0.106	1.00	1.52	1.10	0.112	0.910	1.49	1.02	0.120	0.870	1.37
Digestible Arg diet, %	1.41	0.260	1.03	2.33	1.20	0.110	1.05	1.42	1.13	0.097	0.920	1.27
Digestible Val diet, %	0.870	0.0719	0.720	1.06	0.810	0.0826	0.670	0.955	0.778	0.0642	0.690	0.940
Digestible His, %	0.487	0.0478	0.350	0.600	0.447	0.0649	0.300	0.545	0.451	0.0297	0.410	0.530
Age, d	11.0	3.55	5.00	16.0	20.7	2.21	17.5	24.5	34.9	5.95	28.5	46.0
ADG, g	28.8	6.94	7.36	50.1	61.1	9.88	36.0	81.2	72.3	11.3	46.5	95.1
ADFI, g	42.9	11.9	19.1	74.6	102	10.8	81.6	131	144	18.6	93.5	183
FCR^1^, g/g	1.50	0.273	1.05	3.59	1.69	0.217	1.33	2.49	2.01	0.174	1.58	2.35
Digestible Met intake, g/d	0.184	0.078	0.044	0.449	0.395	0.104	0.197	0.720	0.598	0.168	0.234	1.15
Digestible SAA^2^ intake, g/d	0.308	0.113	0.0915	0.660	0.664	0.155	0.371	1.18	0.998	0.192	0.467	1.59
Synthetic Met dose (%)	0.156	0.117	0.000	0.552	0.147	0.110	0.000	0.460	0.146	0.106	0.000	0.429

1 FCR, feed conversion ratio.

2 SAA, sulfur amino acid (sum of Methionine and Cysteine).

### Growth Function Fitting and Determination of Requirement Parameters

The models for predicting ADG from digestible Met intake along with the selected random effects fitted are graphically presented in Figure 3. The estimated digestible Met requirement parameters *κ* were consistently greater for the quadratic-plateau compared to the linear-plateau model, which were 0.188 ± 0.0101 vs. 0.134 ± 0.0071 g/d, 0.502 ± 0.0432 vs. 0.335 ± 0.0200 g/d and 0.596 ± 0.0389 vs. 0.514 ± 0.0273 g/d for the starter (mean 11.0 d), grower (mean 20.7 d), and finisher broilers (mean 34.9 d), respectively (Table 2). Nonetheless, the LOOIC fit statistic did not consistently favor either of the 2 models. The quadratic-plateau was the best model for the starter data, the linear-plateau for the grower data, and only a marginal difference in LOOIC was observed when fitting the 2 models to the finisher data.

**Table 2:** Linear- and quadratic-plateau model parameters^1^ and leave-one-out information criterion (LOOIC) that were fitted to the three subsets of data using digestible methionine intake as independent variable. Note that the models that were fitted did not account for the type of Met.

Model	α	β	κ	$\sigma_{a}$	$\sigma_{b}$	$\sigma_{k}$	$\sigma_{e}$	LOOIC
Starter subset (*n* = 221)								
Linear-plateau	2.49 (1.28)	210 (11.4)	0.134 (0.0071)	2.57 (0.618)	-	0.0331 (0.00654)	1.29 (0.069)	794
Quadratic-plateau	−1.13 (1.49)	346 (19.4)	0.188 (0.0101)	3.62 (0.730)	-	0.0496 (0.00867)	1.10 (0.0628)	733
Grower subset (*n* = 126)								
Linear-plateau	15.0 (4.05)	143 (15.8)	0.335 (0.0200)	-	25.7 (5.52)	0.0625 (0.0180)	2.61 (0.202)	635
Quadratic-plateau	9.34 (7.23)	230 (41.5)	0.502 (0.0432)	-	31.0 (8.50)	0.0831 (0.0302)	2.90 (0.242)	657
Finisher subset (*n* = 133)								
Linear-plateau	41.9 (3.57)	64.5 (10.5)	0.514 (0.0273)	-	22.0 (4.21)	0.0617 (0.0226)	2.55 (0.183)	656
Quadratic-plateau	16.3 (8.43)	199 (34.3)	0.596 (0.0389	5.98 (1.71)	-	0.113 (0.0288)	2.57 (0.186)	656

1 α is the intercept, β is the slope before κ, which is the breakpoint. $\sigma_{a},\;\sigma_{b},\;\sigma_{k}$ and $\sigma_{e}$ are off diagonal study effects for α, β, κ and the error term.

The models for predicting ADG against digestible SAA intake along with the selected random effects that were fitted are graphically presented in Figure 4. Similar to Met, the SAA requirement parameters *κ* was greater for the quadratic-plateau than the linear-plateau model which was 0.379 ± 0.026 vs. 0.314 ± 0.027 g/d for the starter broilers (Table 3). The quadratic-plateau model fitted the data the best based on LOOIC. The SAA requirement for grower broilers was estimated at 0.932 ± 0.12 g/d using linear-plateau model. The SAA requirement for finisher broilers was estimated at 0.953 ± 0.0477 g/d using linear-plateau model, whereas fitting a quadratic-plateau model to the grower and finisher subsets within the Bayesian hierarchical framework resulted in divergent transition of the Markov chains. In addition, fewer than 5 datapoints were above the 95% credible intervals of the *κ* parameter when fitting these three models, which suggested insufficient datapoints were available for an unbiased estimation of *κ*. In other words, only a few of the records of the grower and finisher subset had SAA intake at or below requirements. Inclusion of covariate such as dietary ME or essential amino acids concentrations did not improve model fit for the three subsets regardless of explanatory variables (digestible Met or SAA intake).

**Table 3:** Linear- and quadratic-plateau model parameters^1^ and leave-one-out information criterion (LOOIC) that were fitted to the three subsets of data using digestible sulfur amino acid intake as independent variable. Note that the models that were fitted did not account for the type of Met.

Model	α	β	κ	$\sigma_{a}$	$\sigma_{b}$	$\sigma_{k}$	$\sigma_{e}$	LOOIC
Starter subset (*n* = 221)								
Linear-plateau	5.52 (2.61)	87.6 (11.2)	0.314 (0.027)	5.99 (1.71)	23.8 (6.04)	0.0956 (0.0191)	0.977 (0.066)	715
Quadratic-plateau	−8.63 (2.37)	217 (12.1)	0.379 (0.026)	8.32 (1.50)	-	0.128 (0.022)	0.904 (0.059)	667
Grower subset (*n* = 126)								
Linear-plateau^2^	24.51 (2.96)	55.15 (5.40)	0.932 (0.12)	**-**	7.16 (1.71)	0.30 (0.147)	2.91 (0.227)	657
Quadratic-plateau^3^								
Finisher subset (*n* = 133)								
Linear-plateau	32.2 (5.46)	45.6 (7.54)	0.953 (0.0477)	-	11.1 (2.10)	0.105 (0.0395)	2.58 (0.186)	661
Quadratic-plateau^4^								

1 α is the intercept, β is the slope before κ, which is the breakpoint. $\sigma_{a},\;\sigma_{b},\;\sigma_{k}$ and $\sigma_{e}$ are off diagonal study effects for α, β, κ and the error term.

2,3,4 Cells are left blank when parameters were not estimable.

### Assessing Effect of Synthetic Methionine Sources

The models for predicting ADG against synthetic Met intake (excluding Met from basal diets) for different phases are shown in Table 4. As stated earlier, linear models were fitted for 4 different datasets of each broiler phase where subsetting was done based on digestible Met or digestible SAA requirement parameter (*κ*) determined using either linear-plateau or quadratic-plateau models. The fitted linear model was extended to evaluate the synthetic Met effect on the slope parameter (β) as shown in Table 5. Again, based on at least 10-point difference in LOOIC value (comparing same model with same observation number from the Tables 4 and 5), the 2 synthetic Met sources did not differ in their performance to fit the broilers’ growth response data. Across broiler phases, no Met effect difference was observed between DL-Met and OH-Met.

**Table 4:** Linear model parameters^1^ and leave-one-out information criterion (LOOIC) that were fitted to the new subset of data using synthetic methionine or sulfur amino acid intake (excluding basal methionine) as independent variable and ADG as dependent variable.

Model	α	β	$\sigma_{a}$	$\sigma_{b}$	$\sigma_{e}$	LOOIC
Starter subset of subset						
Linear (2k_QP,Met_, n = 158)	24.5 (1.42)	47.2 (13.29)	7.2 (1.06)	55.9 (10.68)	1.74 (0.12)	671
Linear (3k_LP,Met_, n = 85)	20.8 (1.60)	147 (64.3)	6.8 (1.22)	308 (74.18)	1.11 (0.11)	300
Linear (4k_QP,SAA_, n = 189)	24.87 (1.36)	34.59 (10.19)	6.84 (1.03)	44.92 (8.42)	2.10 (0.13)	865
Linear (5k_LP,SAA_, n =154)	23.58 (1.59)	42.10 (13.21)	7.23 (1.19)	55.98 (10.94)	1.79 (0.12)	661
Grower subset of subset						
Linear (k_QP,Met_, n = 113)	53.3 (1.85)	70.2 (15.99)	6.7 (1.45)	54.78 (13.74)	3.2 (0.25)	614
Linear (k_LP,Met_, n = 57)	50.7 (2.01)	106.6 (24.12)	7.1 (1.52)	68.6 (19.49)	1.3 (0.17)	216
Linear (k_QP,SAA_, n = ...)^5^						
Linear (k_LP,SAA_, n = 141)	54.60 (1.89)	46.16 (6.60)	6.68 (1.39)	18.78 (5.47)	4.16 (0.28)	826
Finisher subset of subset						
Linear (k_QP,Met_, n = 92)	66.4 (1.20)	56.4 (29.22)	-	97.0 (27.70)	7.9 (0.67)	651
Linear (k_LP,Met_, n =67)	66.82 (2.89)	53.83 (13.59)	11.27 (2.18)	34.29 (19.68)	2.49 (0.39)	337
Linear (k_QP,SAA_, n = ...)^6^						
Linear (k_LP,SAA_, n = 78)	66.10 (1.23)	34.58 (24.85)	-	73.25 (31.58)	8.55 (0.84)	565

1 α is the intercept, β is the slope before κ, which is the breakpoint. $\sigma_{a},\;\sigma_{b},\;\sigma_{k}$ and $\sigma_{e}$ are off diagonal study effects for α, β, κ and the error term.

2 k_QP,Met_: Sub setting was done using quadratic-plateau model using methionine as independent variable.

3 k_LP,Met_: Sub setting was done using linear-plateau model using methionine as independent variable.

4 k_QP,SAA_: Sub setting was done using quadratic-plateau model using sulfur amino acid as independent variable.

5 k_LP,SAA_: Sub setting was done using linear-plateau model using sulfur amino acid as independent variable.

6 Cells are left blank when parameters were not estimable.

**Table 5:** Linear model parameters^1^ and leave-one-out information criterion (LOOIC) that were fitted to the new subset of data using two different forms of synthetic methionine or sulfur amino acid intake (excluding basal methionine) as independent variable and ADG as dependent variable.

Model	α	β1	β2	$\sigma_{a}$	$\sigma_{b}$	$\sigma_{e}$	LOOIC
Starter subset of subset							
Linear (2k_QP,Met_, n = 158)	24.51 (1.46)	46.94 (13.46)	45.78 (13.45)	7.19 (1.09)	55.81 (10.59)	1.74 (0.12)	674
Linear (3k_LP,Met_, n = 85)	20.89 (1.62)	113.45 (51.77)	96.70 (51.46)	6.79 (1.23)	325.7 (78.17)	1.10 (0.11)	299
Linear (4k_QP,SAA_, n = 189)	24.86 (1.40)	35.20 (10.00)	33.19 (10.00)	6.81 (1.00)	45.08 (8.43)	2.10 (0.13)	866
Linear (5k_LP,SAA_, n = 154)	23.62 (1.57)	41.98 (13.36)	40.71 (13.35)	7.22 (1.17)	55.88 (10.35)	1.80 (0.12)	663
Grower subset of subset							
Linear (k_QP,Met_, n = 113)	53.23 (1.9)	73.15 (16.00)	65.48 (16.02)	6.77 (1.45)	54.63 (13.51)	3.2 (0.25)	612
Linear (k_LP,Met_, n = 57)	50.78 (1.93)	118.6 (24.06)	93.92 (23.99)	7.13 (1.54)	68.37 (19.04)	1.03 (0.13)	206
Linear (k_QP,SAA_, n = ...)							
Linear (k_LP,SAA_, n = 141)	54.71 (1.82)	30.00 (7.09)	43.46 (6.81)	6.63 (1.39)	18.92 (5.44)	4.12 (0.28)	825
Finisher subset of subset							
Linear (k_QP,Met_, n = 92)	66.44 (1.17)	53.47 (28.12)	53.63 (28.18)	-	94.82 (27.72)	7.95 (0.70)	652
Linear (k_LP,Met_, n =67)	66.68 (2.80)	53.47 (13.55)	52.22 (14.10)	11.38 (2.11)	32.35 (19.74)	2.54 (0.38)	340
Linear (k_QP,SAA_, n = ...)^6^							
Linear (k_LP,SAA_, n = 78)	66.03 (1.03)	32.70 (23.11)	40.22 (23.48)	-	73.11 (28.88)	8.56 (0.80)	564

1 α is the intercept, β is the slope before κ, which is the breakpoint. $\sigma_{a},\;\sigma_{b},\;\sigma_{k}$ and $\sigma_{e}$ are off diagonal study effects for α, β, κ and the error term.

2 k_QP,Met_: Sub setting was done using quadratic-plateau model using methionine as independent variable.

3 k_LP,Met_: Sub setting was done using linear-plateau model using methionine as independent variable.

4 k_QP,SAA_: Sub setting was done using quadratic-plateau model using sulfur amino acid as independent variable.

5 k_LP,SAA_: Sub setting was done using linear-plateau model using sulfur amino acid as independent variable.

6 Cells are left blank when parameters were not estimable.

## DISCUSSION

Relative efficacy of 2 most common forms of Met (DL-Met vs. OH-Met) has been well-studied and reported performance differences under certain environment and management conditions. Individual studies demonstrated that one form is favored over another under certain conditions, which has created controversy and confusion. Thus, the main goal of this study was to compile up-to-date data from the literature and compare the efficacy of 2 sources of Met using a robust Bayesian meta-analysis approach. We first divided the dataset into starter, grower and finisher age group and then determined Met requirement using either linear or quadratic-plateau models. Most importantly, our final model included the observations below the Met requirements determined using either linear or quadratic-plateau models where previous meta-analysis included all observations within certain dose limits of Met (Sauer et al., 2008; Vazquez-Anon et al., 2006).

### Bayesian Modeling Approach

Recent improvement in computational statistics such as development of programming languages (e.g., WinBUGS and Stan) enables fitting complex models in Bayesian setting (Stan Development Team, 2016). In the current meta-analysis, Bayesian modeling approach was selected because: 1) pre-existing knowledge could be incorporated as prior information in Bayesian model, which helps in estimating the requirement parameter κ by restricted it to the range of the independent variable, viz. digestible Met and SSA intake and 2) the Bayesian approach facilitates fitting of complex models with large number of random variances (Sorensen et al., 2016). Additionally, Bayesian approach provides reliable estimate of the variance components with relatively small dataset which is often not possible using non-Bayesian approach (Bates et al., 2015). Meta-analysis often warrants powerful statistical approach because meta-analysis using frequentist approach for non-linear regression (Sauer et al., 2008) or multi-exponential regression (Hoehler et al., 2005) models might face difficulty to converge due to lack of suitable starting values for the parameters. In that sense, Bayesian approach does not require any starting values for model convergence. In Bayesian modeling, posterior distribution is influenced by the prior information and data. In general, priors are set as flat priors so that the posterior distribution is mostly influenced by the data (Sorensen et al., 2016). In the current study, minimally informative priors for most parameters were used, thus posterior distribution was allowed to be determined mainly by the data used in this analysis. Estimation of between study heterogeneity is also crucial for the meta-analysis which might affect inferences. In traditional meta-analysis approach, choosing a suitable heterogeneity estimator is challenging and might add arbitrariness to the inferences (Veroniki et al., 2016). However, in Bayesian framework, accounting for uncertainty is straightforward and even estimates are valid for small dataset (Pappalardo et al., 2020). Therefore, compared to traditional frequentist approach, Bayesian meta-analysis approach used in the current study is better, more powerful and advantageous to deal with between study heterogeneity with a careful choice of priors even with limited number of studies and missing values (Röver, 2020; Pappalardo et al., 2020).

### Determination of Requirement Parameters

In this meta-analysis, both linear- and quadratic-plateau models were used to determine the requirements for Met and SAA with ADG as the response variable. Previous studies used both ADG and FCR as the response variable (e.g., Sauer et al., 2008). The choice of ADG as the response variable in this study was based on exploratory analysis of scatter plots, which showed very weak relationship between independent variables and FCR in comparison to the strong relationship between independent variables and ADG. Furthermore, the use of ratio variable (e.g., FCR) in general is difficult to make practical inference from the results. Previous meta-analysis conducted by Sauer et al. (2008) and Vazquez-Anon et al. (2006) used Met dose as the explanatory variable. In contrast, in this study we found a better fit of the model using digestible Met or SAA intake instead of dose as explanatory variable.

Growth response of birds fed different Met sources depends on the Cys levels in the diet for starter phase, but it was not the case for grower or finisher phase of broiler (Pillai et al., 2006). This interaction between Met source and Cys level had been reported by Dilger and Baker (2008). These authors observed that excess Cys in Met deficient diet led to decrease in feed intake and adverse effect on OH-Met growth response. Therefore, we also used the digestible SAA intake as the explanatory variable instead of Met to determine if it makes any difference between Met sources. In the case of SAA requirement, the grower dataset contained fewer data points for broilers fed SAA at or below requirements. Therefore, all data points were included in the subset of grower dataset for further analysis. Using digestible SAA as the response variable also did not make the difference in ADG of broilers at any phases fed 2 different sources of Met. It is important to note that we also observed a very large variability of ADG for birds fed control diets because Met concentration in control diets varied widely across studies. Further analysis revealed that variation in Met concentration in basal diets explained approximately 95% variability of ADG for birds within control group when Met concentration was regressed against ADG (data not shown).

### Synthetic Methionine and Biological Efficacy

This meta-analysis using current literature data with Bayesian approach demonstrated that growth response (ADG) of broiler was not influenced by form of the synthetic Met precursors. In agreement with our study, Vazquez-Anon et al. (2006) also reported a similar effect of DL-Met and OH-Met on broiler performance using multilinear regression approach. However, the meta-analysis by Sauer et al. (2008) found a significant difference in ADG between DL-Met and OH-Met using a nonlinear mixed modeling approach. They also reported a lower relative biological efficacy of 81% for OH-Met in comparison to DL-Met for ADG whereas relative biological efficacy of OH-Met in comparison to DL-Met did not differ statistically in our study. This discrepancy could be partly explained by the differences in modeling approach and data sources. Our study included data from studies published between 1985 and 2019 where greater than 50% (20 of 39) of the studies included were conducted or published between 2007 and 2019. In contrast, the meta-analysis by Sauer et al. (2008) included studies published between 1983 and 2006 Sauer et al. (2008). also modeled the growth as response to the dose in percent of active supplemental Met whereas in the current study, the models were applied using the supplemental Met intake as explanatory variable. This latter variable takes into account the feed intake which has been shown by several authors to vary according to the dietary SAA level (Vazquez-Anon et a., 2017; Zhao et al., 2018). Additionally, the analyzed active supplemental Met dose (%) is also rarely given in most of the studies; thus, using supplemental Met dose (%) does not capture the variability during feed manufacture and of feed consumption. Thus, the genetic and management improvement of birds might have affected the growth performance response to Met dose response in previous studies (Vazquez-Anon et al., 2006). The inclusions of more recent studies and the use of daily digestible Met intake instead of dose as explanatory variable also helped us to avoid this confounding effect to some extent. Age of bird might have played another role because the data in this study was divided into 3 age groups whereas Sauer et al. (2008) observed that age had significant effects on the ADG intercept of the model and thus add age as covariate in the model (Sauer et al., 2008). In conclusion, using powerful Bayesian meta-analysis approach including the most recent studies, no significant statistical difference was detected in ADG in response to the most common dietary synthetic methionine forms (i.e. DL-Met and OH-Met) at or below the requirement. Thus, favoring one form of Met over another on the basis of growth performances (ADG in this case) may depend on the availability, cost, ease of inclusion in the diet, and choice of the producer. Additionally, some other factors and conditions such as reducing N excretions (Kim et al., 2014), supporting animal production during heat stress (Dibner et al., 1992) and acting as an antioxidant (Li et al., 2014) lead producers to favor one form over the other.

## Appendix Supplementary materials

Image, application 1
