# Supplementary material for: Evaluating growth response of broiler chickens fed diets supplemented with synthetic DL-methionine or DL-hydroxy methionine: a meta-analysis
Source: Poult Sci. 2022 Feb 2;101(5):101762. doi: 10.1016/j.psj.2022.101762 (PMC8917292; doi:10.1016/j.psj.2022.101762)
Supplement: Supplementary file 1 [file mmc1.docx]

**APPENDIX**

**List of articles and report included in the database**

1. Abdel-Maksoud, A., F. Yan, S. Cerrate, Z. Wang, and P. W. Waldroup. 2010. Effect of arginine levels and source and level of methionine on performance of broiler 0 to 18 days of age. Int. J. Poult. Sci. 9:14-20.
2. Agostini, P. S., P. Dalibard, Y. Mercier, P. van der Aar, and J. D. van der Klis. 2015. Comparison of methionine sources around requirement level using a methionine efficacy method in 0 to 28 days old broilers. Poult. Sci. 95:560-569.
3. Balnave, D., J. Hayat, and J. Brake. 1999. Dietary arginine: lysine ratio and methionine activity at elevated environmental temperatures. J. Appl. Poult. Res. 8:1-9.
4. Bunchasak, C., T. Sooksridang, and R. Chaiyapit. 2006. Effect of adding methionine hydroxy analogue as methionine source at the commercial requirement recommendation on production performance and evidence of ascites syndrome of male broiler chicks fed corn-soybean based. Int. J. Poult. Sci. 5:744-752.
5. Cadirci, S. and S. Koncagul. 2014. Possible effects of delivering methionine to broilers in drinking water at constant low and high environmental temperatures. Ital. J. Anim. Sci. 13:93-97.
6. Conde-Aguilera J. A., J. C. G Cholet, M. Lessire, Y. Mercier, S. Tesseraud, and J. van Milgen. 2016. The level and source of free-methionine affect body composition and breast muscle traits in growing broilers. Poult. Sci. 95:2322–2331.
7. Daenner, E. and W. Bessei1. 2003. Influence of supplementation with liquid DL-Methionine hydroxy analogue-free acid (Alimet) or DL-Methionine on performance of broilers. J. Appl. Poult. Res. 12:101-105.
8. Del Vesco, A. P., E. Gasparino, A. R. Oliveira Neto, S. E. Guimarães, S. M. Marcato, and D. M. Voltolini. 2013. Dietary methionine effects on IGF-I and GHR mRNA expression in broilers. Genet. Mol. Res. 12: 6414-6423.
9. Del Vesco, A P., E. Gasparino, A. R. Oliveira Neto,  R. M. Rossi, M. A. M. Soares, and S. C. C. Silva. 2013. Effect of methionine supplementation on mitochondrial genes expression in the breast muscle and liver of broilers. Livest. Sci. 151:284-291.
10. Drazbo, A., K. Kozlowski, L. C. Siwiecka, A. Sobczak, P. Kwiatkowski, and A. Lemme. 2015. Effect of different dietary level of DL-methionine and calcium salt of DL-2-hydroxy-4-(methyl) butanoic acid on the growth performance, carcass yield and meat quality of broiler chickens. Eur. Poult. Sci. 79:1-15.
11. Esteve-Garcia, E., and L. L. Llaurado. 1997. Performance, breast meat yield and abdominal fat deposition of male broiler chickens fed diets supplemented with DL-methionine or DL-methionine hydroxy analogue free acid. Br. Poult. Sci. 38:397-404.
12. Garlich, J. D. 1985. Response of broiler to dl-Met hydroxyl analogue free acid, dl-Met, and l-Met. Poult. Sci. 64:1541-1584.
13. Kim, D., B. K. An, S. Oh, M. C. Keum, S. Lee, J. S. Um, T. Ayasan, and K. W. Lee. 2019. Effects of different methionine sources on growth performance, meat yield and blood characteristics in broiler chickens. J. Appl. Anim. Res. 47:230-235.
14. Leite, R. S., J. S. R. Rocha, , B. C. Michell, E. A. Lara, E. A. Ornelas, S. V. Cançado, and , N.C. Baião. 2009. Efeitos de planos nutricionais e de fontes de metionina sobre o desempenho, rendimento e composição de carcaças de frangos de corte. Arquivo Brasileiro de Medicina Veterinária e Zootecnia, 61:120-1127.
15. Lemme, A., D. Hoehler, J. J. Brennan, and P. F. Mannion. 2002. Relative effectiveness of methionine hydroxy analog compared to dl-methionine in broiler chickens. Poult. Sci. 81:838-845.
16. Liu, Y. L., G. L. Song,  G. F. Yi,  Y. Q. Hou,  J. W. Huang,  M. Vazquez-Anon,  and C. D. Knight. 2006. Effect of supplementing 2-hydroxy-4-(methylthio) butanoic acid and dl-methionine in corn–soybean–cottonseed meal diets on growth performance and carcass quality of broilers. Asian-Austral. J. Anim. Sci. 19:1197-1205.
17. Liu, Y. L., G. F. Yi, G. L. Song, Y. Q. Hou, J. W. Huang, M. Vazquez-Anon, and C. D. Knight. 2007. Impact of feeding 2-hydroxy-4- (methylthio) butanoic acid and DL-methionine supplemented maize-soybean-rapeseed meal diets on growth performance and carcase quality of broilers. Br. Poult. Sci. 48(2):190-197.
18. Lu, J. J., C. W. Huang, and R. G. R. Chou. 2003. The effects of DL-Methionine hydroxyl analogue on growth performance, contents of serum amino acids and activities of digestive proteases in broilers. Asian-Austral. J. Anim. Sci. 16:714-718.
19. Mandal, A. B., A. V. Elangovan, and T. S. Johri. 2004. Comparing bioefficacy of liquid DL-methionine hydroxy analogue free acid with DL-methionine in broiler chickens. Asian-Austral. J. Anim. Sci. 17:102-108.
20. Morales, B. E., G. E. Avila, and N. Wagner. 1991. Comparison of two sources of methionine in diets for fattening chickens. Vet. Mex. 22:267-271.
21. Payne, R. L., A. Lemme, H. Seko, Y. Hashimoto, H. Fujisaki, J. Koreleski, S. Swiatkiewicz, W. Szczurek, and H. Rostagno. 2006. Bioavailability of methionine hydroxy analoguefree acid relative to dl-methionine in broilers. Anim. Science Journal, 77:427-439.
22. Pillai, P. B., A. C. Fanatico, M. E. Blair, and J. L. Emmert. 2006. Homocysteine remethylation in broilers fed surfeit choline or betaine and varying levels of methionine from eight to twenty-two days of age. Poult. Sci. 85:1729-1736.
23. Rakangtong, C., and C. Bunchasak. 2010. Effects of dietary energy and methionine sources on productive performance and carcass yield in broiler chickens. Kaset. J-Nat. Sci. 44:574-581.
24. Ribeiro, A. M. L., F. Dahlkeand, and A. M. Kessler. 2005. Methionine sources do not affect performance and carcass yield of broilers fed vegetable diets and submitted to cyclic heat stress. Braz. J. Poult. Sci. 7:159-164.
25. Salary, J., M. Kalantar, F. Dashtbin, and H. R. Hemati-Matin. 2015. ALIMET® (liquid methionine hydroxy analogue) in broiler chicken diets: immunity system, microflora population, and performance. Arch. de Zootec**. 64**:57-62.
26. Sangali, C. P., L. D. G. Bruno, R. V. Nunes, A. R. O. Neto, P. C. Pozza, T. M. M. Oliveira, R. Frank, and R. A. Schöne. 2014. Bioavailability of different methionine sources for growing broilers. Braz. J. Anim. Sci. 43:140-145.
27. Sangali, C. P., L. D. G. Bruno, R. V., Nunes, A. R. O. Neto, P. C. Pozza, J. R. Henz, F. C. N. Giacobbo and E. Berwanger. 2015. Bioavailability of different methionine sources for broilers from 1 to 21 days old. Cienc. Investig. Agrar. 42:35-43.
28. Schutte, J. B., J. de Jong, W. Smink, and M. Pack. 1996. Biological efficacy of DL-methionine hydroxy analog-free acid compared to DL-methionine in broiler chicks as determined by performance and breast meat yield. Agribiol. Res. 49:74-82.
29. Summers, J. D., S. Blackman, and S. Leeson. 1987. Assay for estimating the potency of various methionine-active sources. Poult. Sci. 66:1779-1787.
30. Thomas, O. P., C. Tamplin, S. D. Crissey, E. Bossard, and A. Zuckerman. 1991. An evaluation of methionine hydroxy analog free acid using a nonlinear (exponential) bioassay. Poult. Sci. 70: 605-610.
31. [Ullrich, C](https://www.ncbi.nlm.nih.gov/pubmed/?term=Ullrich%20C%5BAuthor%5D&cauthor=true&cauthor_uid=31752397)., M. [Langeheine,](https://www.ncbi.nlm.nih.gov/pubmed/?term=Langeheine%20M%5BAuthor%5D&cauthor=true&cauthor_uid=31752397) R. [Brehm,](https://www.ncbi.nlm.nih.gov/pubmed/?term=Brehm%20R%5BAuthor%5D&cauthor=true&cauthor_uid=31752397) V. [Taube,](https://www.ncbi.nlm.nih.gov/pubmed/?term=Taube%20V%5BAuthor%5D&cauthor=true&cauthor_uid=31752397) M. [Rosillo-Galera,](https://www.ncbi.nlm.nih.gov/pubmed/?term=Rosillo%20Galera%20M%5BAuthor%5D&cauthor=true&cauthor_uid=31752397) K. [Rohn,](https://www.ncbi.nlm.nih.gov/pubmed/?term=Rohn%20K%5BAuthor%5D&cauthor=true&cauthor_uid=31752397) J.  [Popp,](https://www.ncbi.nlm.nih.gov/pubmed/?term=Popp%20J%5BAuthor%5D&cauthor=true&cauthor_uid=31752397) and C. [Visscher](https://www.ncbi.nlm.nih.gov/pubmed/?term=Visscher%20C%5BAuthor%5D&cauthor=true&cauthor_uid=31752397). 2019. Influence of Different Methionine Sources on Performance and Slaughter Characteristics of Broilers. [Animals](https://www.ncbi.nlm.nih.gov/pubmed/31752397), 9(11):984.
32. Wang, Y., X. Yin, D. Yin, Z. Lei, T. Mahmood, and J. Yuan. 2019. Antioxidant response and bioavailability of methionine hydroxy analog relative to DL-methionine in broiler chickens. Anim. Nutr. 5:241-247.
33. Vázquez-Añón, M., R. González-Esquerra, E. Saleh, T. Hampton, S. Ritcher, J. Firman, and C. D. Knight. 2006. Evidence for 2-hydroxy-4(methylthio) butanoic acid and dl-methionine having different dose responses in growing broilers. Poult. Sci. 85:1409-1420.
34. Viana, M. T. S., L. F. T. Albino, H. S. Rostagno, S. L. T. Barreto, D. C. O. Carvalho, and P. C. Gomes. 2009. Methionine sources and levels in broiler chick diets. Braz. J. Anim. Sci. 38: 1751-1756.
35. Yao, J. H, S. Q. Li, L. L. Zhong, S. X. Huang, W. J. Zhang, and H. B. Xi. 2006. The relative effectiveness of liquid methionine hydroxy analogue compared to DL-methionine in broilers. Asian-Austral. J. Anim. Sci. 19:1026-1032.
36. Zhang, S., B. Saremi, E. R. Gilbert, and E. A. Wong. 2017. Physiological and biochemical aspects of methionine isomers and a methionine analogue in broilers. Poult. Sci. 96:425-439.
37. Zeitz, J. O., S. Mohrmann, L. Fehse, E. Most, A. Helmbrecht, B. Saremi, and K. Eder. 2018. Tissue and plasma antioxidant status in response to dietary methionine concentration and source in broilers. J. Anim. Physiol. Anim. Nutr. 102:999-1011.
38. Zelenka, J., J. Heger, V. Machander, M. Wiltafsky, and M. Lestak. 2015. Bioavailability of liquid methionine hydroxy analogue-free acid relative to DL-methionine in broilers. Acta Univ. Agric. Silvic. Mendel. Brun. 168:1513–1520.
39. Zou, L., D. Wang, J. Liu, Y. Bai, Z. Liang, and T. Zhang. 2015. Effects of DL-2-hydroxy-4-(methylthio) butanoic acid on broilers at different dietary inclusion rates. Br. Poult. Sci. 56:337-344.
